# Manifold alteration between major depressive disorder and healthy control subjects using dynamic mode decomposition in resting-state fMRI data

**DOI:** 10.3389/fpsyt.2024.1288808

**Published:** 2024-01-30

**Authors:** Hidenori Endo, Shigeyuki Ikeda, Kenichiro Harada, Hirotaka Yamagata, Toshio Matsubara, Koji Matsuo, Yoshinobu Kawahara, Okito Yamashita

**Affiliations:** ^1^ Center for Advanced Intelligence Projects, RIKEN, Tokyo, Japan; ^2^ Department of Computational Brain Imaging, Advanced Telecommunications Research Institute International (ATR) Neural Information Analysis Laboratories, Kyoto, Japan; ^3^ Faculty of Engineering, University of Toyama, Toyama, Japan; ^4^ Division of Neuropsychiatry, Department of Neuroscience, Yamaguchi University Graduate School of Medicine, Yamaguchi, Japan; ^5^ Department of Psychiatry, Faculty of Medicine, Saitama Medical University, Saitama, Japan; ^6^ Graduate School of Information Science and Technology, Osaka University, Osaka, Japan

**Keywords:** resting-state fMRI, dynamic mode decomposition, major depressive disorder, manifold, density ratio estimation

## Abstract

**Background:**

The World Health Organization has reported that approximately 300 million individuals suffer from the mood disorder known as MDD. Non-invasive measurement techniques have been utilized to reveal the mechanism of MDD, with rsfMRI being the predominant method. The previous functional connectivity and energy landscape studies have shown the difference in the coactivation patterns between MDD and HCs. However, these studies did not consider oscillatory temporal dynamics.

**Methods:**

In this study, the dynamic mode decomposition, a method to compute a set of coherent spatial patterns associated with the oscillation frequency and temporal decay rate, was employed to investigate the alteration of the occurrence of dynamic modes between MDD and HCs. Specifically, The BOLD signals of each subject were transformed into dynamic modes representing coherent spatial patterns and discrete-time eigenvalues to capture temporal variations using dynamic mode decomposition. All the dynamic modes were disentangled into a two-dimensional manifold using t-SNE. Density estimation and density ratio estimation were applied to the two-dimensional manifolds after the two-dimensional manifold was split based on HCs and MDD.

**Results:**

The dynamic modes that uniquely emerged in the MDD were not observed. Instead, we have found some dynamic modes that have shown increased or reduced occurrence in MDD compared with HCs. The reduced dynamic modes were associated with the visual and saliency networks while the increased dynamic modes were associated with the default mode and sensory-motor networks.

**Conclusion:**

To the best of our knowledge, this study showed initial evidence of the alteration of occurrence of the dynamic modes between MDD and HCs. To deepen understanding of how the alteration of the dynamic modes emerges from the structure, it is vital to investigate the relationship between the dynamic modes, cortical thickness, and surface areas.

## 1 Introduction

The World Health Organization has reported that approximately 300 million individuals suffer from the mood disorder known as major depressive disorder (MDD). MDD gives rise to psychological symptoms, such as despondent moods and negative cognitions, as well as physical symptoms, such as sleep disturbances and fatigue in mild cases, and even suicide in severe cases (1). Neurotransmitter reuptake inhibitors, such as selective serotonin reuptake inhibitors and transcranial magnetic stimulation through electrical stimulation, have been employed in the treatment of MDD (2–4). Although these treatments are effective, there are patients whose depressive symptoms improve only partially or not at all (5). Therefore, the mechanisms underlying MDD need to be elucidated.

Non-invasive measurement techniques have been utilized to reveal the mechanism of MDD, with resting-state functional magnetic resonance imaging (rsfMRI) being the predominant method (6). To evaluate dynamic changes in blood oxygenation level-dependent (BOLD) signals using rsfMRI, static functional connectivity (sFC), dynamic functional connectivity (dFC), and energy landscape (EL) were employed as indices to portray the dynamics of whole-brain networks. sFC captures the static relationships of spontaneous fluctuations that represent correlations over the entire duration (7, 8), whereas dFC captures time-resolved spontaneous fluctuations in which functional connectivity (FC) changes over a short time (9–11). Evaluation of the static and dynamic relationships of spontaneous fluctuations in the whole-brain network has revealed that MDD exhibits abnormal connections in FC, such as the default mode network (DMN), control executive network (CEN), and salience network (SN) when compared with healthy controls (HCs) (12–16). Analyzing sFC involves calculating the correlation between two independent regions for all pairs (17). Even if a pair of regions is not directly structurally interconnected, their sFC can exhibit a strong correlation if both regions receive input from a third region (18). Hence, it is imperative to simultaneously represent the dynamics of whole-brain networks based on neural activity across multiple regions. This is where EL emerges, which utilizes a pairwise maximum entropy model to represent the dynamics of the whole-brain network in terms of the activity within each region and the interactions between two or more regions (19). Moreover, by defining the functional network between subjects in terms of energy, it is possible to evaluate the transition from one stable state to another through the unstable states. Notably, MDD tends to sink to specific states, and it is difficult to transition from one stable state to another compared to HCs (20). Although EL excels in stability analysis across subjects, some issues require prior assignment of a functional network to each region and binarization of BOLD signals. In common with sFC, dFC, and EL, analyzing components of the BOLD signal above 0.1 Hz is a challenging problem. Therefore, in terms of interactions across multiple regions, a methodology is required to evaluate the sinking into specific states under conditions free from functional network assignment and binarization.

The dynamic mode decomposition (DMD) is a data-driven and equation-independent approach for analyzing fluid dynamics (21). DMD calculates eigenvectors and corresponding eigenvalues of the approximate linear transformation expressing the time evolution of multidimensional time-series data. Eigenvectors were called dynamic modes (DMs) representing coherent spatial patterns and the corresponding eigenvalues were called discrete-time eigenvalues representing the frequency and time evolution such as growth and decay. In other words, multiple coherent DMs coexist at a certain time in multidimensional time-series data and corresponding temporal characteristics are identified. EL analysis assigns a functional network to each region, binarizes the BOLD signal, fits it with a Boltzmann distribution, determines relationships between activity patterns and energy, and assigns one state on EL at a certain time in multidimensional time-series data (22). Here, since the BOLD signals exhibit wave superposition, it is necessary to analyze stability under conditions where multiple states coexist at a certain time. DMD was successful and recent studies have applied DMD to BOLD signals, a type of fluid that exhibits nonlinear spatiotemporal changes (23–26). This study applied DMD to the BOLD signals across all frequency bands of HCs and MDD. Subsequently, the spatial patterns, frequencies, and temporal changes across all subjects were analyzed in terms of stability.

Analysis of a large dataset of psychiatric disorders based on rsfMRI (27) using DMD revealed that the number of DMs associated with MDD decreased in visual networks (VN) and SN, while it increased in DMN and sensory-motor networks (SMN) when compared to HCs. Interestingly, DMs’ differences between MDD and HCs were identified not only within the 0.01–0.1 Hz range in standard rsfMRI analysis but also extending beyond 0.1 Hz. Applying t-distribution stochastic neighbor embedding (t-SNE) (28) to DMs enables the disentangling of the intricate curved surfaces spanned by DMs into a two-dimensional manifold, allowing for the evaluation of stability across subjects. Subsequently, DMs resembling resting-state networks (RSNs) were identified by evaluating the probability density ratio between HCs and MDD using a two-dimensional manifold. The amplitudes of the DMs resembling the VN and SN were similar to the spatial patterns associated with cortical thickness and surface area abnormalities in MDD (29).

## 2 Materials and methods

**Figure 1:**
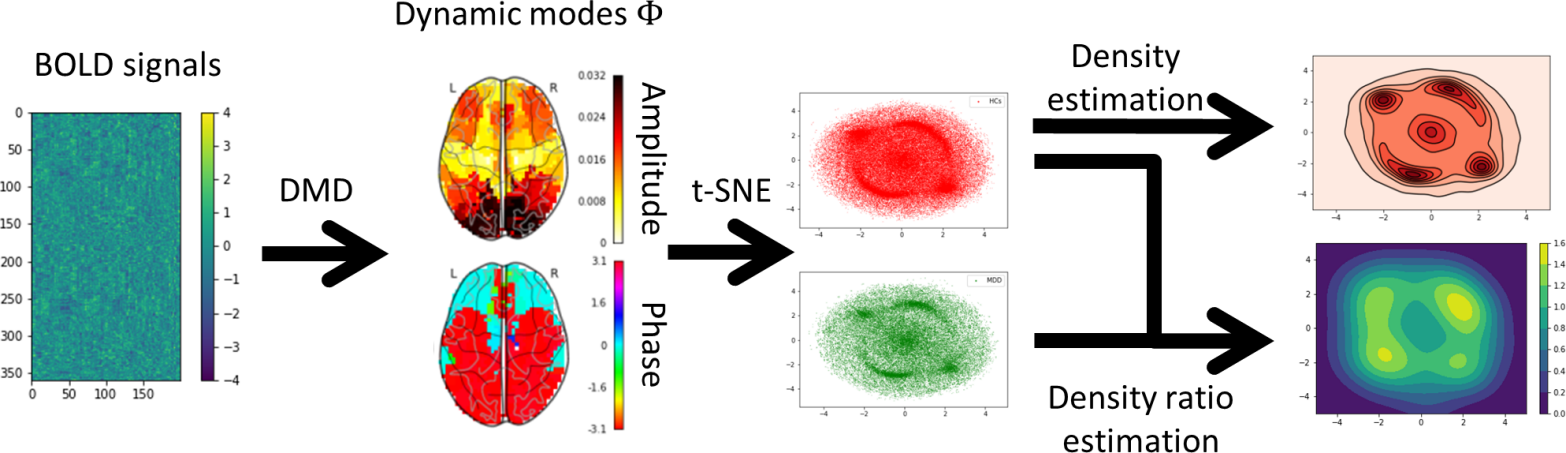
Overview of the analysis procedure. First, each subject’s blood oxygenation level-dependent (BOLD) signals were extracted using Glasser’s 360 regions of interest (ROI). Second, the BOLD signals were decomposed into dynamic modes (DMs) and discrete-time eigenvalues using the one-stacked time-delay coordinates dynamic mode decomposition (tdcDMD). Third, all DMs were disentangled into the two-dimensional manifold using t-distributed stochastic neighbor embedding (t-SNE). Fourth, density estimation was performed to visualize the features that major depressive disorder (MDD) sink into the specific DMs compared to healthy controls (HCs). Finally, density ratio distributions between HCs and MDD were calculated using relative unconstrained least-squares importance fitting (RuLSIF).

In this study, we applied DMD to BOLD signals and devised a method for extracting DMs based on the probability density ratio between HCs and MDD on two-dimensional manifolds using t-SNE (**Figure 1**). First, the BOLD signals of each subject were transformed into DMs representing coherent spatial patterns and discrete-time eigenvalues to capture temporal variations using DMD. Second, all the DMs were disentangled into a two-dimensional manifold using t-SNE. Finally, density estimation and density ratio estimation were applied to the two-dimensional manifolds after the two-dimensional manifold was split based on the HCs and MDD. The results revealed that MDD tended to sink into specific DMs in contrast to HCs.

### 2.1 Dataset

We used the Japanese Strategic Research Program for the Promotion of Brain Science (SRPBS) dataset (27) (https://bicr.atr.jp/decnefpro/data/), along with additional datasets obtained from various projects. **Supplementary Table 1** describes the protocols at each site, and **Supplementary Table 2** describes the subject information at each site.

The datasets were collected from the Center of Innovation at Hiroshima University (COI) and the University of Tokyo (UTO), Hiroshima Kajikawa Hospital (HKH), Hiroshima Rehabilitation Center (HRC), Hiroshima University Hospital (HUH), and Yamaguchi University (UYA). COI and UTO follow the unified protocol but HKH, HRC, HUH, and UYA follow non-unified protocols. The total number of HCs and MDD was 543 and 302, respectively, with Beck Depression Inventory-II (BDI-II) scores of 7.5 ± 6.3 and 28.1 ± 10.5, respectively.

### 2.2 BOLD signals preprocessing

BOLD signals were preprocessed using fMRIPrep version 1.0.8 (http://fmriprep.readthedocs.io/en/1.0.8/workflows.html) (30). The first 10 s of the data were discarded to allow for T1 equilibration. The preprocessing steps included slice-timing correction, realignment, coregistration, distortion correction using a field map, segmentation of T1-weighted structural images, normalization to Montreal Neurological Institute space, and spatial smoothing with an isotropic Gaussian kernel of 6 mm full width at half maximum. “Fieldmap-less” distortion correction was performed for the test dataset due to the lack of field map data.

### 2.3 Preprocess of ROI time series for DMD

It is necessary to mitigate the effects of the protocols and physiological noise. BOLD signal extraction was performed using Glasser’s 360 regions of interest (ROI) (31), which excluded the cerebellum and contained little white matter.

Nilearn’s NiftiLabelsMasker function (https://nilearn.github.io/stable/index.html) was used for the BOLD signal extraction. Detrending was applied to eliminate long-term variations, and BOLD signals were normalized using z-scores to mitigate the effects of the protocols. When analyzed using the DMD, the frequencies were computed for each DM. Therefore, band-pass filtering was not applied.

Confounding factors must be removed when extracting BOLD signals. The fit _transform function was applied to remove confounding factors for the 12 regression parameters (six motion parameters, average signals over the whole brain, and five anatomical CompCor components).

### 2.4 One-stacked time-delay coordinates DMD

BOLD signals were decomposed into DMs and discrete-time eigenvalues. Time-delay coordinates DMD (tdcDMD) is a method used for decomposing standing waves into spatiotemporal patterns with high accuracy (21); tdcDMD was performed using the dmd.py function in the DMD toolbox (https://github.com/erichson/DMDpack). As described in a previous study (26), the BOLD signals of each subject were converted into DMs. As shown in Equation 1, the BOLD signal matrix ***X*** was composed of rows representing the number of ROI, ${\text{N}}_{\text{roi}}$ and columns representing the number of measurements, ${\text{N}}_{\text{T}}$ .


(1) $$\begin{array}{c} X=\left[\begin{array}{cccc} x_{1} & x_{2} & \cdots & x_{N_{T}} \end{array}\right], \end{array}$$

where $x_{k}\left(\in R^{Nroi}\right)$ represents the BOLD signals at time *k*. The following matrices were constructed from the BOLD signal matrix ***X*** as shown in Equations 2, 3.


(2) $$\begin{array}{c} X_{1}=\left[\begin{array}{cccc} x_{1} & x_{2} & \cdots & x_{N_{T}-1} \end{array}\right], \end{array}$$

(3) $$\begin{array}{c} X_{2}=\left[\begin{array}{cccc} x_{2} & x_{3} & \cdots & x_{N_{T}} \end{array}\right], \end{array}$$

where $X_{2}$ represents the matrix with $X_{1}$ shifted back one observation. Subsequently, $x_{k+1}$ was stacked on $x_{k}$ as shown in Equations 4, 5.


(4) $$\begin{array}{c} X_{1aug}=\left[\begin{array}{cccc} x_{1} & x_{2} & \cdots & x_{N_{T}-2}\\ x_{2} & x_{3} & \cdots & x_{N_{T}-1} \end{array}\right], \end{array}$$

(5) $$\begin{array}{c} X_{2aug}=\left[\begin{array}{cccc} x_{2} & x_{3} & \cdots & x_{N_{T}-1}\\ x_{3} & x_{4} & \cdots & x_{N_{T}} \end{array}\right], \end{array}$$

$X_{2aug}$ was predicted using $X_{1aug}$ so $X_{2aug}\approx AX_{1aug}$ .


(6) $$\begin{array}{c} A=X_{2aug}X_{1aug}^{†}, \end{array}$$

where the dagger represents the generalized inverse. Singular value decomposition was applied to $X_{1aug}$ .


(7) $$\begin{array}{c} X_{1aug}=U\Sigma V^{*}, \end{array}$$

where U, Σ, and V represent the left singular, singular value, and right singular matrices of $X_{1aug}$ , respectively. As shown in Equation 8, the matrix *A* is rewritten by substituting Equation 7 into Equation 6.


(8) $$\begin{array}{c} A=X_{2aug}V\Sigma^{-1}U^{*}, \end{array}$$

the proper orthogonal decomposition was applied to ***A*.**


(9) $$\begin{array}{c} \tilde{A}=U^{*}AU=U^{*}X_{2aug}V\Sigma^{-1}, \end{array}$$

then eigen decomposition was applied to $\tilde{A}$ .


(10) $$\begin{array}{c} \tilde{A}W=W\Lambda , \end{array}$$

where W and Λ represent the eigenvector and eigenvalue matrices of $\tilde{A}$ , respectively. $X_{2aug}V\Sigma^{-1}$ was multiplied from the left in Equation 10 and Equation 9 was substituted into Equation 10.


(11) $$\begin{array}{c} AX_{2aug}V\Sigma^{-1}W=X_{2aug}V\Sigma^{-1}W\Lambda ,\; \end{array}$$

where $\tilde{A}$ is the similar matrix of A , so they have the same eigenvalue matrix Λ but different eigenvector matrices. In comparing Equations 10, 11, $X_{2aug}V\Sigma^{-1}W$ can be regarded as the eigenvector matrix of ***A***. Finally, the eigen decomposition of ***A*** was reconstructed using W and Λ and the dynamic mode matrix Φ was calculated as shown in Equation 12.


(12) $$\begin{array}{c} \Phi =X_{2aug}V\Sigma^{-1}W, \end{array}$$

the *i*-th column of Φ , which we denote by $\phi_{i}\left(\in {\mathbb{C}}^{2N_{roi}}\right)$ , is the *i*-th eigenvector of A . The *i*-th diagonal element of Λ , which we denote by $\lambda_{i}\left(\in \mathbb{C}\right)$ , is the i-th eigenvalue of A . The phase and amplitude of $\lambda_{i}$ mean the frequency and decay rate of the corresponding mode. The frequency $f_{i}$ corresponding to the dynamic mode $\phi_{i}$ and the eigenvalue $\lambda_{i}$ is described as following Equation 13.


(13) $$\begin{array}{c} f_{i}=\frac{imag\left(\ln\left(\lambda_{i}\right)\right)}{2\pi \Delta t}, \end{array}$$

where Δt , ln(·) and imag(·) represent the temporal resolution in each protocol, natural logarithm, and the imaginary part of a complex number.

### 2.5 Two-dimensional manifold with t-SNE

When analyzed using the DMD, pairs of DMs with identical amplitudes but antiphases emerged. Moreover, DMs representing brain states describe intricate curved surfaces in a multidimensional space. In a previous study (26), the modified K-means clustering algorithm was applied to DMs and treated DMs with identical amplitudes and antiphases. However, this approach failed to disentangle intricate curved surfaces in a multidimensional space. Hence, this study employed t-SNE (28) to disentangle the intricate curved surfaces spanned by DMs.

The initial 360 rows, which are inherently independent of the 720 rows of the DMs, were used to employ a one-stacked tdcDMD. Subsequently, the DMs were separated into their real and imaginary components, stacked together, and applied to t-SNE. When t-SNE was applied to all DMs of both HCs and MDD, the perplexity varied from 30 to 10,000. A value of 2,000 was visually selected to achieve maximum separation between peaks within the two-dimensional manifold while keeping random_state fixed. The sklearn.manifold.TSNE function in Python was employed, with all parameters set to their default values except perplexity and random_state.

### 2.6 Kernel density estimation

It is crucial to select the optimal perplexity at which the peaks within the two-dimensional manifold achieve maximum separation. Hence, we separated the peaks by performing a kernel density estimation on a two-dimensional manifold. The formula for estimating the probability density ρ at a given point y , estimated from points $x_{\text{i}}\left(i=1,\;2,\;\ldots ,\text{ n}\right)$ of DMs on the two-dimensional manifold is expressed as following Equation 14:


(14) $$\begin{array}{c} \rho \left(y\right)=\sum_{i}^{n}K\left(y-x_{i};h\right), \end{array}$$

where kernel *K* is the Gaussian kernel and bandwidth h is set to the Scotts factor. Scipy.stats.gaussian_kde function in Python was used (32).

### 2.7 Kernel density ratio estimation

The probability density was estimated using kernel density estimation on the two-dimensional manifolds obtained by applying t-SNE. Consequently, the distinction between HCs and MDD was revealed as a different balance in the proportion of DMs rather than the emergence of unknown DMs. Hence, we estimated the probability density ratio between HCs and MDD using a relatively unconstrained least-squares importance fitting (RuLSIF) (33). In terms of estimation accuracy, it is more precise to directly estimate the density ratio between HCs and MDD than to indirectly estimate the density ratio by estimating HCs and MDD’s densities separately and dividing HCs and MDD’s densities. To improve the estimation accuracy, various methods have been developed to directly estimate the density ratio without going through the density estimation process. RuLSIF was chosen for this study because its Python code is publicly available and its calculation speed is fast.

The optimal parameters were automatically selected in the range of coefficient α=0 , the regularization parameter η=0.10, 0.09, …, 0.01, and Gaussian kernel width σ=1.2, 1.0, 0.8 . RuLSIF was performed using the toolbox (https://github.com/hoxo-m/densratio_py).

To estimate the density ratio of the area where the HCs’ density was higher than the MDD’s density, the HCs’ manifold was used as the denominator, and the MDD’s manifold was used as the numerator. To estimate the density ratio for the area where the MDD’s density was higher than the HCs’ density, the MDD’s manifold was used as the denominator, and the HCs’ manifold was used as the numerator.

### 2.8 Plotting dynamic modes, histogram of frequency, and discrete-time eigenvalues greater than 95% significance level

Kernel density ratio estimation was used to calculate the probability density ratio between HCs and MDD. However, the specific regions exhibiting significant differences in terms of density ratio between HCs and MDD remain unknown. To solve this problem, permutation tests were performed to clarify areas higher than the 95% significance level and to plot the mean amplitude and phase of the DMs, a histogram of frequency, and discrete-time eigenvalues within the significant areas.

First, we randomized the labels of the HCs and MDD in a two-dimensional manifold. Second, with fixed parameters (α, σ, η)=(0, 1.0, 0.01 ), RuLSIF was performed to calculate the maximum peak value, repeating this process 100 times. Third, we applied the density-based spatial clustering of applications with noise (DBSCAN) (34) to cluster points within areas that exhibited maximum peak values higher than the 95th percentile. Finally, we plotted the mean amplitudes and phases of the DMs, frequency histograms, and discrete-time eigenvalues λ associated with each cluster. For the density ratios $p_{\text{MDD}}\left(x\right)/p_{\text{HCs}}\left(x\right)$ and $p_{\text{HCs}}\left(x\right)/p_{\text{MDD}}\left(x\right)$ , the DBSCAN parameters were set as (eps, min samples)=(1, 100) and (0.15, 300) , respectively. Points that were not assigned to a cluster were excluded.

## 3 Results

### 3.1 Applying t-SNE, density estimation, and density ratio estimation to the DMs

**Figure 2:**
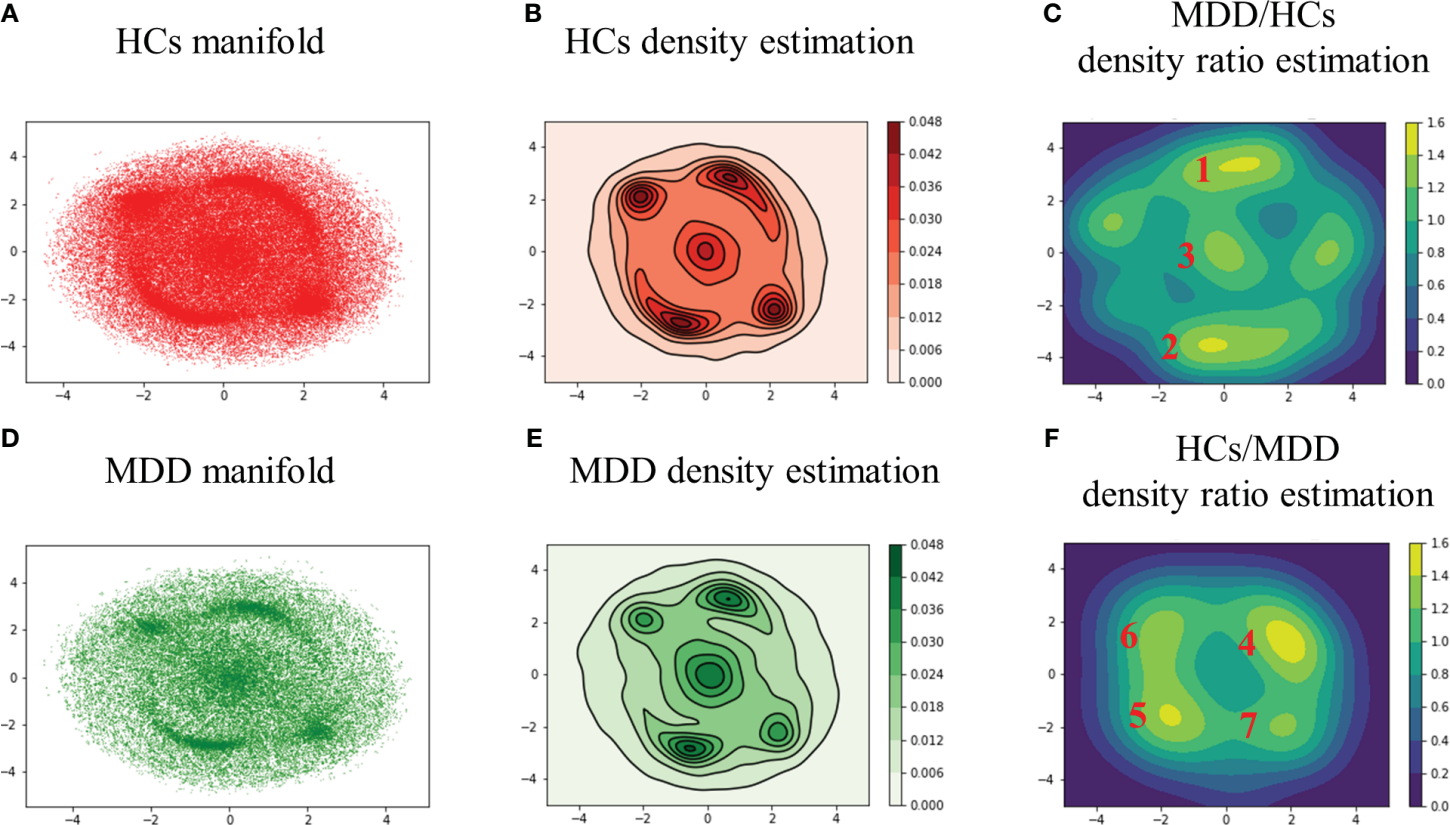
Two-dimensional manifolds of HCs **(A)** and MDD **(B)** with t-SNE, kernel density estimation of HCs **(C)** and MDD **(D)**, and density ratio distribution estimated by relative unconstrained least-squares importance fitting (RuLSIF) in the case of MDD/HCs **(E)** and HCs/MDD **(F)**. The points on the two-dimensional manifold indicate DMs **(A, B)**. The curved lines on the density estimation indicate contour lines **(C, D)**. The red numbers indicate the peak number. In the MDD/HCs case, the peaks located at the far left and far right were not assigned numbers due to their lack of significance at the 95% confidence level **(E, F)**. MDD/HCs shows increased DMs in MDD, and HCs/MDD shows reduced DMs in MDD.

First, the two-dimensional manifold was calculated by applying t-SNE to all DMs across all subjects and was visualized after separating the HCs and MDD (**Figure 2A**: HCs, B: MDD). Second, the perplexity was varied from 30 to 10,000 and consequently set to 2,000 to maximally separate the peaks in the two-dimensional manifold. Finally, kernel density estimation was performed to clarify the distribution features exhibited by the two-dimensional manifold (**Figure 2C**: HCs, D: MDD).

In the HCs, the peaks displayed a relatively uniform distribution (**Figure 2C**). Conversely, in the MDD group, the peaks exhibited a bias toward the upper right, lower left, and central areas (**Figure 2D**). In other words, MDD tended to sink more into specific DMs than HCs. In addition, the edge of the MDD manifold appeared slightly wider than that of the HCs manifold at the elliptical periphery. To assess these features, density ratio estimation was performed by applying RuLSIF to the two-dimensional manifolds.

### 3.2 DM’s features in the clusters

The density ratio was calculated using HCs as the denominator and MDD as the numerator (**Figure 2E**). Similarly, the density ratio was calculated using the MDD as the denominator and HCs as the numerator (**Figure 2F**). The colored bars represent the value of the density ratios. For parameter search, α=0 , the regularization parameter η varied from 0.10 to 0.01, and the Gaussian kernel width σ took values of 1.2, 1.0, and 0.8. As a result, η=0.01 and σ=1.0 were selected. After performing the density ratio estimation, it was necessary to determine the significant areas. Therefore, a permutation test was performed with α=0, η=0.01, and σ=1.0 . The labels of HCs and MDD across all DMs were shuffled, and density ratio estimation was applied to calculate the maximum peak value 100 times (**Supplementary Figure S1**). Subsequently, areas above the 95th percentile of the maximum peak value were calculated (**Supplementary Figures S2A, B**) and clustered using DBSCAN (**Supplementary Figures S2C, D**).

**Figure 3:**
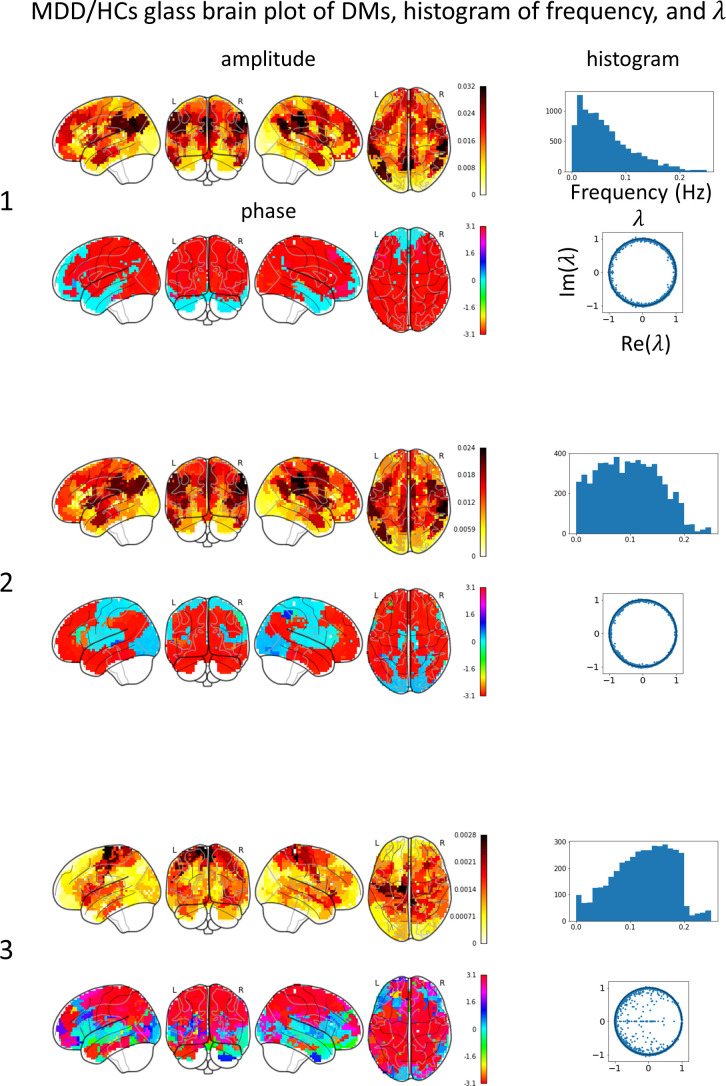
Mean DMs’ amplitude, phase, histogram of frequency, discrete-time eigenvalue λ in each MDD/HCs cluster. The left numbers correspond to the peak numbers in **Figure 2**. DM1 resembles the default mode network (DMN), has a low frequency, and is stable. DM2 resembles DMN, has a flat frequency, and is stable. DM3 resembles a sensory-motor network (SMN), has high frequency, and tends to converge.

**Figure 4:**
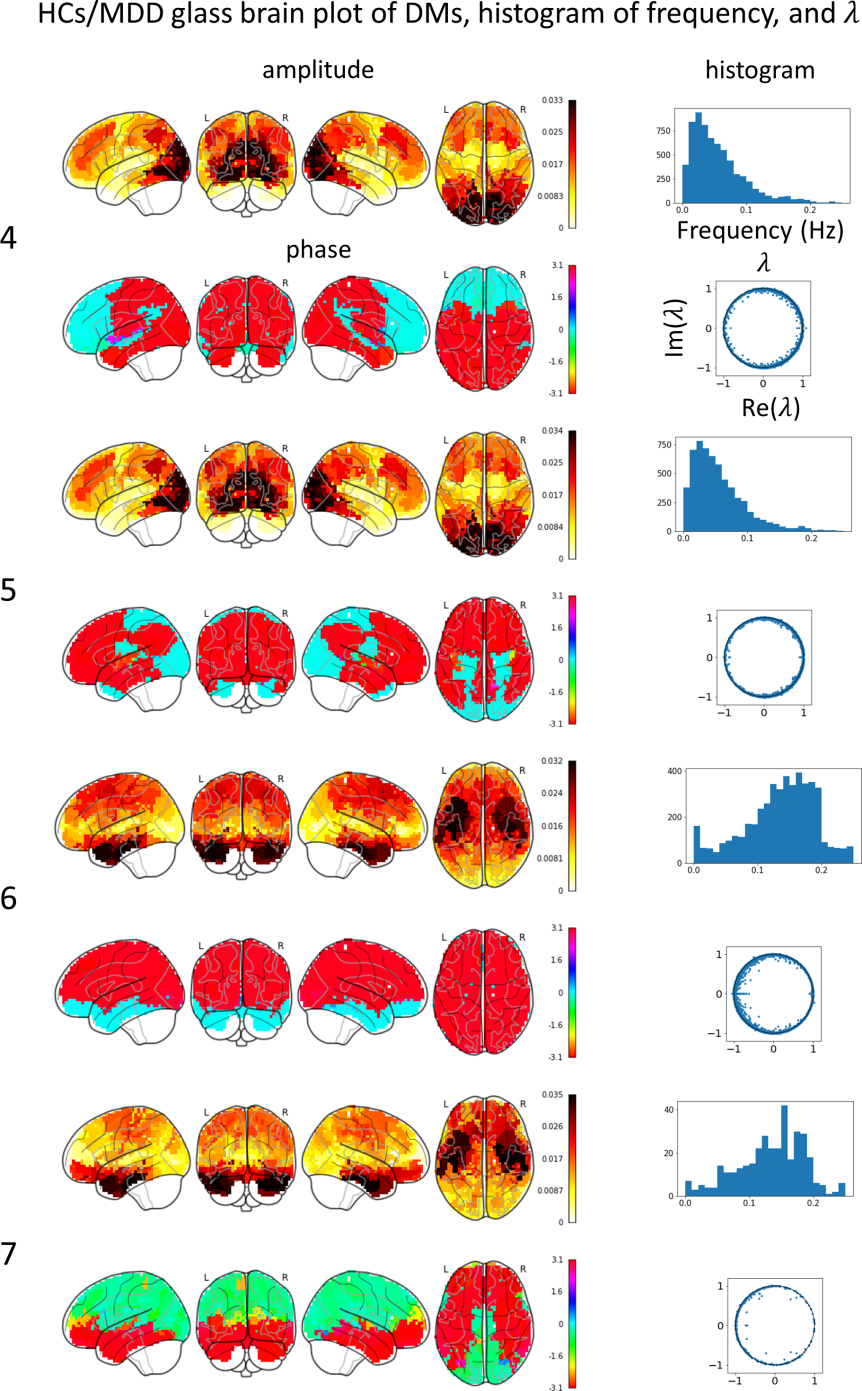
Mean DM’s amplitude, phase, histogram of frequency, discrete-time eigenvalue λ in each HCs/MDD cluster. The left numbers correspond to the peak numbers in **Figure 2**. DM4 resembles a visual network (VN), has a low frequency, and is stable. DM 5 resembles a VN, has a low frequency, and is stable. DM6 resembles a salience network (SN), has a high frequency, and is stable. DM 7 resembles an SN, has a high frequency, and is stable.

Glass brain plots depicting the amplitude and phase of the mean DMs, histograms of frequency, and discrete-time eigenvalues within clusters in the MDD/HCs (**Figure 3**) and HCs/MDD (**Figure 4**) cases are presented. Because DMs appear in pairs with modes of identical amplitude and an anti-phase relationship, DMs at symmetric locations are paired (**Figure 2E** 1-2, **Figure 2F** 4-5, and 6-7).

In the MDD/HCs case, the glass brain plots of DM1 and DM2 were similar to those of DMN. The discrete-time eigenvalues were distributed along the unit circle, indicating stability in DM1 and DM2. The glass brain plots of DM3 were similar to those of the SMN. The discrete-time eigenvalues were relatively numerous inside the unit circle, indicating not only stability but also convergence in DM3. Additionally, because both the DMN and SMN were concurrently active in DM2, the frequency histogram was likely to show an intermediate distribution between the distributions in DM1 and DM3.

In the HCs/MDD case, the glass brain plots of DM4 and DM5 were similar to those of the VN. The discrete-time eigenvalues were distributed along the unit circle, indicating stability in DM4 and DM5. The histogram of the frequency showed a peak at approximately 0.03 Hz. The glass-brain plots of DM6 and DM7 were similar to those of the SN. The discrete-time eigenvalues were distributed along the unit circle, indicating stability in DM6 and DM7. The histogram of the frequency showed a peak at approximately 0.15 Hz. The small number of DMs in DM7 likely resulted in a negative bias of the phase and scattering of the frequency histogram.

## 4 Discussion

We devised a methodology for estimating brain-state stability across subjects by applying DMD to BOLD signals; t-SNE was applied to the DMs to disentangle the intricate curved surface spanned by the DMs into a two-dimensional manifold (**Figure 2**). Density ratio estimation was then performed on the two-dimensional manifolds of HCs and MDD (**Figures 2E, F**). Consequently, it was revealed that MDD did not cause the emergence of unknown DMs distinct from HCs but sank into specific DMs, such as DM1, DM2, and DM3.

In machine learning using DMD, there are two important aspects of comparing HCs and MDD. One is interpretability in terms of physiology and the other is classification performance for biomarker. Therefore, individual-level classification between HCs and MDD was performed to demonstrate usability to the biomarker development (**Supplementary Figure S6**). As a result, when evaluated using 10-fold cross-validation (**Supplementary Figure S7**), the balanced accuracy (Bacc) was slightly better than that in the previous study (12) using sFC (**Supplementary Figure S8**).

### 4.1 Dynamic modes and cortical abnormalities of MDD

The spatial patterns of reduced DMs corresponded to the patterns observed in the cortical thickness and surface area abnormalities (29). Specifically, DM6 and DM7 exhibited spatial patterns similar to the reductions in cortical thickness observed in adult MDD, whereas DM4 and DM5 displayed spatial patterns resembling the reductions in cortical surface area observed in adolescent MDD. Therefore, the reduction in DM4, DM5, DM6, and DM7 levels plays a key role in elucidating the mechanisms of MDD.

Widespread abnormalities have been discovered in MDD, from microscopic phenomena such as the genome and molecular pathways to macroscopic phenomena such as BOLD signals. Microscopic mutations are environmentally influenced, promote synaptic degeneration with inflammation, lead to mesoscopic neuronal firing abnormalities weighted by the neurotransmitter map, and result in macroscopic abnormalities, such as BOLD signals (35–39). Related to mesoscopic phenomena, some abnormalities are observed in the reuptake of neurotransmitters, such as serotonin, dopamine, norepinephrine, and GABA (40–42) resulting in neurotransmitter concentrations in plasma metabolism (43). Related to macroscopic phenomena, MDD exhibits reduced cortical thickness and surface area compared with HCs (29). As if to connect these two different scale phenomena, both the cortical abnormalities and receptor maps share similar spatial patterns (44, 45). These combined abnormalities likely resulted in sinking into specific DMs, such as DM1, DM2, and DM3. Hence, if a subject transitions from HCs to MDD, it is plausible that MDD would submerge into these particular DMs alongside reductions in cortical thickness and surface area, as well as neurotransmitter reuptake abnormalities.

As a first step in integrating multiple pieces of information that reflect different aspects of MDD, it is vital to investigate the relationship between alterations in stability based on DMs and reductions in cortical thickness and surface area using large datasets. In a comprehensive study on white matter alterations in HCs and MDD, fractional anisotropy was found to be decreased in adult MDD but not significantly different in adolescent MDD compared to HCs (46). Conversely, adolescent MDD exhibited decreased cortical surface areas, particularly in regions such as the orbitofrontal cortex and lateral occipital cortex, when compared to HCs (29). Therefore, in addition to examining the structural connectivity based on the fiber structure in the white matter, it is essential to consider stability measures based on reduced cortical surface areas in both HCs and MDD. Notably, sFC can be well explained (approximately 0.9) by geometric modes (GMs) derived from the cortical geometric structure in HCs (47), suggesting that GMs could serve as a valuable stability indicator based on brain structure.

The integration of multiple indicators will be effective in psychiatric care. A combination of temporally stable trait biomarkers and temporally variable state biomarkers is necessary for early diagnosis and intervention using mechanism-based treatments (48). Therefore, structural connectivity and GMs, as temporally stable trait biomarkers, are employed as criteria for assessing stability. Additionally, DMs serve as temporally variable state biomarkers for evaluating the current cortical stability. The integration of the stability associated with cortical structural and geometric alterations and BOLD signals may shed light on previously unknown mechanisms underlying MDD.

### 4.2 Inconsistency with the previous studies

In MDD, negative emotions are associated with increased activity in the DMN (49) and motor impairment is associated with slow gait and slumped posture (50). Consequently, DM1 and DM2, resembling the DMN, probably emerged for experiencing negative emotions, and DM3, resembling the SMN, probably emerged for experiencing movement difficulties.

In the EL-based method (20), non-melancholic MDD tended to sink into the left CEN, whereas melancholic MDD tended to sink into both the left CEN and dorsal DMN states. In contrast, in the DMD-based method, the MDD sinks into brain states resembling the DMN and SMN. These differences can be attributed to the following three factors. First, the binarization process affects the results. In the DMD-based method, the strong amplitudes of all DMs, except for DM3, were approximately 0.03 in regions associated with the DMN, VN, and SN, and medium amplitudes were approximately 0.01 in regions associated with the SMN. In contrast, the strongest amplitudes of DM3 were associated with the SMN, but the amplitude value was only 0.003, which is approximately 1/10 compared with the other DMs. Conversely, the EL-based method requires the binarization of BOLD signals after functional network assignment to a specific region. This binarization process may have led to an outcome in which regions with amplitudes smaller than the average were considered inactive. Second, the larger number of subjects in our study may lead to more robust results than the previous study. This study included 845 subjects, whereas the previous study included 262 subjects. Lastly, regarding the subtype of MDD, this study did not differentiate between non-melancholic and melancholic MDD, whereas previous studies analyzed these subtypes separately. These methodological discrepancies and different numbers of subjects may account for the sinking into different states between the DMD- and EL-based methods.

In a large dataset study using the sFC (13), hypoconnectivities were observed within the SMN and SN, as well as between the SMN, SN, dorsal attention network (DAN), and VN in MDD. However, no significant differences were found between the DMN and fronto-parietal networks (FPN). In contrast, this study identified abnormalities in the DMN, SMN, VN, and SN but no abnormalities in the DAN. A previous study using the same dataset showed that there were only a few abnormal FCs related to the DAN and many abnormal FCs related to the DMN (12). It is worth noting that the DMN and DAN exhibit an inverse correlation, wherein DAN activation leads to DMN suppression (51). Therefore, it is possible that the subjects in this study activated the DMN, while those in the larger dataset study used an sFC-activated DAN (13).

### 4.3 Relationships among DMs’ spatial pattern, histogram of frequency, and discrete-time eigenvalue

The amplitude of DM3 exhibited a spatial pattern resembling that of the SMN and was approximately 0.003, which was approximately 1/10 smaller than the amplitudes of the other DMs. The amplitudes in DM6 and DM7 were stronger in the SN and slightly stronger in the SMN than in the other DMs. The amplitudes of DM1 and DM2 were stronger in the DMN and slightly stronger in the SMN. Consequently, the SMN tended to appear more frequently in conjunction with other networks. Furthermore, the observation that the SMN tended to co-occur with low-frequency DM1 and DM2, as well as high-frequency DM6 and DM7, suggests that DM3 transmitted information across a broad range of frequencies, resulting in a smoother frequency distribution compared to the other DMs.

A comparative study investigating empirical and simulated sFC and dFC proposed that SMN serves as a driver of cortical dynamics (52). The SMN probably exhibits weak amplitudes and a wide frequency range across all DMs because of its role as a driver in cortical dynamics.

### 4.4 Limitation of the current method

t-SNE was employed to disentangle the intricate curved surfaces spanned by the DMs and analyze the inter-subject stability. However, the method used in the study encountered two problems. First, the computation time was considerable, requiring approximately one week to apply t-SNE to approximately 160,000 DMs, search for the optimal perplexity, estimate the density ratio using RuLSIF, and calculate the clusters based on permutation tests. Consequently, the search for optimal parameters was limited to perplexity during the t-SNE. It is noteworthy that t-SNE encompasses additional parameters, including the early exaggeration factor, learning rate, angle, and random_state, which also influence the manifold. These parameters were determined using a heuristic method (53) in the sklearn.manifold. Second, memory usage has become a serious concern as increasing the perplexity of t-SNE consumes up to approximately 100 GB. To analyze larger datasets, alternative methods such as deep learning or other approaches need to be developed.

When performing rsfMRI, some subjects rarely lacked BOLD signals in the cerebellum. Additionally, BOLD signals from the white matter often contain significant noise. To avoid these issues, the stability analysis between HCs and MDD in this study utilized Glasser’s 360 ROI, which excludes the cerebellum and predominantly consists of gray matter. Therefore, to analyze intersubject stability using ROI that includes the cerebellum and white matter, alternative methods such as deep learning or other approaches need to be developed instead of this method.

**Supplementary Figure S5** shows the normalized number of DMs for each protocol, which was obtained by dividing the number of DMs in the cluster by the total number of DMs in the protocol. The COI and UTO employed a unified protocol, whereas HKH, HUH, HRC, and UYA employed independent protocols. However, the normalized histogram of site in COI tended to be closer to UYA and HKH, while the normalized histogram of site in UTO tended to be closer to HUH and HRC. **Supplementary Table 1** showed that Siemens manufactured COI, UYA, and HKH while GE manufactured UTO, HUH, and HRC. The inter-protocol differences in DM6 and DM7 were more dependent on manufacturers such as Siemens and GE than on protocol unification (**Supplementary Table 1**). In a previous study on physiological noise (54), the approximately 0.2 Hz component of BOLD signals was affected by respiration. In addition, the FD values of DM 6 and 7 were higher than those of other DMs, as shown in **Supplementary Figures S3**, **S4**. However, the Bacc in the case of using all frequencies was higher than that of using 0.01–0.08 Hz, as shown in **Supplementary Figure S8**. Thus, as in previous research (26), there are more spontaneous fluctuations representing cortical dynamics than noise associated with respiration, head movement, and manufacture.

## Data availability statement

The data analyzed in this study is subject to the following licenses/restrictions: The SRPBS dataset except for UYA can be downloadable from the DecNef Project Brain Data Repository. UYA is available to only our research group. Requests to access these datasets should be directed to https://hbm.brainminds-beyond.jp/data.html.

## Ethics statement

The studies involving humans were approved by Yamaguchi University [H23-153 and H25-85]. The studies were conducted in accordance with the local legislation and institutional requirements. The participants provided their written informed consent to participate in this study.

## Author contributions

HE: Conceptualization, Formal analysis, Writing – original draft, Investigation, Methodology, Software, Visualization. SI: Conceptualization, Investigation, Methodology, Software, Writing – review & editing. KH: Data curation, Writing – review & editing. HY: Data curation, Writing – review & editing. TM: Data curation, Writing – review & editing. KM: Data curation, Writing – review & editing. YK: Methodology, Writing – review & editing. OY: Conceptualization, Funding acquisition, Investigation, Methodology, Project administration, Writing – review & editing.

## References

[1] Otte C Gold SM Penninx BW Pariante CM Etkin A Fava M. Major depressive disorder. Nat Rev Dis Primers (2016) 2:1–20. doi: 10.1038/nrdp.2016.65 27629598

[2] Moncrieff J Cooper RE Stockmann T Amendola S Hengartner MP Horowitz MA. The serotonin theory of depression: a systematic umbrella review of the evidence. Mol Psychiatry (2022) 2022:1–14. doi: 10.1038/s41380-022-01661-0 PMC10618090 35854107

[3] Somani A Kar SK. Efficacy of repetitive transcranial magnetic stimulation in treatment-resistant depression: the evidence thus far. Gen Psychiatr (2019) 32:100074. doi: 10.1136/GPSYCH-2019-100074 PMC6738665 31552384

[4] Cipriani A Furukawa TA Salanti G Chaimani A Atkinson LZ Ogawa Y. Comparative efficacy and acceptability of 21 antidepressant drugs for the acute treatment of adults with major depressive disorder: a systematic review and network meta-analysis. Lancet (2018) 391:1357–66. doi: 10.1016/S0140-6736(17)32802-7 PMC5889788 29477251

[5] Marwaha S Palmer E Suppes T Cons E Young AH Upthegrove R. Novel and emerging treatments for major depression. Lancet (2023) 401:141–53. doi: 10.1016/S0140-6736(22)02080-3 36535295

[6] Lee MH Smyser CD Shimony JS. Resting-state fMRI: a review of methods and clinical applications. AJNR Am J Neuroradiol (2013) 34:1866–72. doi: 10.3174/AJNR.A3263 PMC4035703 22936095

[7] Fox MD Greicius M. Clinical applications of resting state functional connectivity. Front Syst Neurosci (2010) 4:19/BIBTEX. doi: 10.3389/FNSYS.2010.00019/BIBTEX 20592951 PMC2893721

[8] Biswal B Zerrin Yetkin F Haughton VM Hyde JS. Functional connectivity in the motor cortex of resting human brain using echo-planar mri. Magn Reson Med (1995) 34:537–41. doi: 10.1002/MRM.1910340409 8524021

[9] Filippi M Spinelli EG Cividini C Agosta F. Resting state dynamic functional connectivity in neurodegenerative conditions: A review of magnetic resonance imaging findings. Front Neurosci (2019) 13:657/BIBTEX. doi: 10.3389/FNINS.2019.00657/BIBTEX 31281241 PMC6596427

[10] Hutchison RM Womelsdorf T Allen EA Bandettini PA Calhoun VD Corbetta M. Dynamic functional connectivity: Promise, issues, and interpretations. Neuroimage (2013) 80:360–78. doi: 10.1016/J.NEUROIMAGE.2013.05.079 PMC3807588 23707587

[11] Menon SS Krishnamurthy K. A comparison of static and dynamic functional connectivities for identifying subjects and biological sex using intrinsic individual brain connectivity. Sci Rep (2019) 9:1–11. doi: 10.1038/s41598-019-42090-4 30952913 PMC6450922

[12] Yamashita A Sakai Y Yamada T Yahata N Kunimatsu A Okada N. Generalizable brain network markers of major depressive disorder across multiple imaging sites. PloS Biol (2020) 18:e3000966. doi: 10.1371/JOURNAL.PBIO.3000966 33284797 PMC7721148

[13] Javaheripour N Li M Chand T Krug A Kircher T Dannlowski U. Altered resting-state functional connectome in major depressive disorder: a mega-analysis from the PsyMRI consortium. Trans Psychiatry (2021) 11:1–9. doi: 10.1038/s41398-021-01619-w PMC8497531 34620830

[14] Kaiser RH Whitfield-Gabrieli S Dillon DG Goer F Beltzer M Minkel J. Dynamic resting-state functional connectivity in major depression. Neuropsychopharmacology (2016) 41:1822–30. doi: 10.1038/NPP.2015.352 PMC4869051 26632990

[15] Drysdale AT Grosenick L Downar J Dunlop K Mansouri F Meng Y. Resting-state connectivity biomarkers define neurophysiological subtypes of depression. Nat Med (2016) 23:28–38. doi: 10.1038/nm.4246 27918562 PMC5624035

[16] Dinga R Schmaal L Penninx BWJH van Tol MJ Veltman DJ van Velzen L. Evaluating the evidence for biotypes of depression: Methodological replication and extension of. NeuroImage Clin (2019) 22:101796. doi: 10.1016/J.NICL.2019.101796 30935858 PMC6543446

[17] Zuo XN Ehmke R Mennes M Imperati D Castellanos FX Sporns O. Network centrality in the human functional connectome. Cereb Cortex (2012) 22:1862–75. doi: 10.1093/CERCOR/BHR269 21968567

[18] Adachi Y Osada T Sporns O Watanabe T Matsui T Miyamoto K. Functional connectivity between anatomically unconnected areas is shaped by collective network-level effects in the macaque cortex. Cereb Cortex (2012) 22:1586–92. doi: 10.1093/CERCOR/BHR234 21893683

[19] Watanabe T Hirose S Wada H Imai Y Machida T Shirouzu I. A pairwise maximum entropy model accurately describes resting-state human brain networks. Nat Commun (2013) 4:1–10. doi: 10.1038/ncomms2388 PMC3660654 23340410

[20] Regonia PR Takamura M Nakano T Ichikawa N Fermin A Okada G. Modeling heterogeneous brain dynamics of depression and melancholia using energy landscape analysis. Front Psychiatry (2021) 12:780997/BIBTEX. doi: 10.3389/FPSYT.2021.780997/BIBTEX 34899435 PMC8656401

[21] Kutz JN Brunton SL Steven L Brunton BW Procor JL. Dynamic mode decomposition: data-driven modeling of complex systems. SIAM (2016). doi: 10.1137/1.9781611974508

[22] Ezaki T Watanabe T Ohzeki M Masuda N. Energy landscape analysis of neuroimaging data. Phil. Trans. R. Soc. A., vol. 375. (2017). doi: 10.1098/RSTA.2016.0287 PMC5434078 28507232

[23] Brunton BW Johnson LA Ojemann JG Kutz JN. Extracting spatial–temporal coherent patterns in large-scale neural recordings using dynamic mode decomposition. J Neurosci Methods (2016) 258:1–15. doi: 10.1016/J.JNEUMETH.2015.10.010 26529367

[24] Shiraishi Y Kawahara Y Kawahara Y Yamashita O Yamashita O Fukuma R. Neural decoding of electrocorticographic signals using dynamic mode decomposition. J Neural Eng (2020) 17:036009. doi: 10.1088/1741-2552/ab8910 32289756

[25] Casorso J Kong X Chi W Van De Ville D Yeo BTT Liégeois R. Dynamic mode decomposition of resting-state and task fMRI. Neuroimage (2019) 194:42–54. doi: 10.1016/J.NEUROIMAGE.2019.03.019 30904469

[26] Ikeda S Kawano K Watanabe S Yamashita O Kawahara Y. Predicting behavior through dynamic modes in resting-state fMRI data. Neuroimage (2022) 247:118801. doi: 10.1016/J.NEUROIMAGE.2021.118801 34896588

[27] Tanaka SC Yamashita A Yahata N Itahashi T Lisi G Yamada T. A multi-site, multi-disorder resting-state magnetic resonance image database. Sci Data (2021) 8:1–15. doi: 10.1038/s41597-021-01004-8 34462444 PMC8405782

[28] Van Der Maaten LJP Hinton GE. Visualizing high-dimensional data using t-sne. J Mach Learn Res (2008) 9:2579–605.

[29] Schmaal L Hibar DP Sämann PG Hall GB Baune BT Jahanshad N. Cortical abnormalities in adults and adolescents with major depression based on brain scans from 20 cohorts worldwide in the ENIGMA Major Depressive Disorder Working Group. Mol Psychiatry (2016) 22:900–9. doi: 10.1038/mp.2016.60 PMC5444023 27137745

[30] Esteban O Markiewicz CJ Blair RW Moodie CA Isik AI Erramuzpe A. fMRIPrep: a robust preprocessing pipeline for functional MRI. Nat Methods (2018) 16:111–6. doi: 10.1038/s41592-018-0235-4 PMC6319393 30532080

[31] Glasser MF Coalson TS Robinson EC Hacker CD Harwell J Yacoub E. A multi-modal parcellation of human cerebral cortex. Nature (2016) 536:171–8. doi: 10.1038/nature18933 PMC4990127 27437579

[32] Scott DW. Multivariate density estimation: Theory, practice, and visualization. John Wiley & Sons, 2nd ed. (2015). doi: 10.1002/9781118575574.

[33] Yamada M Suzuki T Kanamori T Hachiya H Sugiyama M. Relative density-ratio estimation for robust distribution comparison. Neural Comput (2013) 25:1324–70. doi: 10.1162/NECO_A_00442 23547952

[34] Ester M Kriegel H-P Sande J Xu X. A Density-Based Algorithm for Discovering Clusters in Large Spatial Databases with Noise, KDD-96. AAAI press (1996). pp. 226–31.

[35] Nir Y Fisch L Mukamel R Gelbard-Sagiv H Arieli A Fried I. Coupling between neuronal firing rate, gamma LFP, and BOLD fMRI is related to interneuronal correlations. Curr Biol (2007) 17:1275–85. doi: 10.1016/J.CUB.2007.06.066 17686438

[36] Fries GR Saldana VA Finnstein J Rein T. Molecular pathways of major depressive disorder converge on the synapse. Mol Psychiatry (2022) 28:284–97. doi: 10.1038/s41380-022-01806-1 PMC9540059 36203007

[37] Ramirez-Mahaluf JP Roxin A Mayberg HS Compte A. A computational model of major depression: the role of glutamate dysfunction on cingulo-frontal network dynamics. Cereb Cortex (2017) 27:660–79. doi: 10.1093/CERCOR/BHV249 PMC5939208 26514163

[38] Kringelbach ML Cruzat J Cabral J Knudsen GM Carhart-Harris R Whybrow PC. Dynamic coupling of whole-brain neuronal and neurotransmitter systems. Proc Natl Acad Sci USA (2020) 117:9566–76. doi: 10.1073/pnas.1921475117 32284420 PMC7196827

[39] Li J Seidlitz J Suckling J Fan F Ji GJ Meng Y. Cortical structural differences in major depressive disorder correlate with cell type-specific transcriptional signatures. Nat Commun (2021) 12:1–14. doi: 10.1038/s41467-021-21943-5 33712584 PMC7955076

[40] Conio B Martino M Magioncalda P Escelsior A Inglese M Amore M. Opposite effects of dopamine and serotonin on resting-state networks: review and implications for psychiatric disorders. Mol Psychiatry 2019 25:1 (2019) 25:82–93. doi: 10.1038/s41380-019-0406-4 30953003

[41] Moret C Briley M. The importance of norepinephrine in depression. Neuropsychiatr Dis Treat (2011) 7:9. doi: 10.2147/NDT.S19619 21750623 PMC3131098

[42] Luscher B Shen Q Sahir N. The GABAergic deficit hypothesis of major depressive disorder. Mol Psychiatry (2011) 16:383. doi: 10.1038/MP.2010.120 21079608 PMC3412149

[43] Pan JX Xia JJ Deng FL Liang WW Wu J Yin BM. Diagnosis of major depressive disorder based on changes in multiple plasma neurotransmitters: a targeted metabolomics study. Trans Psychiatry (2018) 8:1–10. doi: 10.1038/s41398-018-0183-x PMC6039504 29991685

[44] Hansen JY Shafiei G Markello RD Smart K Cox SML Nørgaard M. Mapping neurotransmitter systems to the structural and functional organization of the human neocortex. Nat Neurosci (2022) 25:1569–81. doi: 10.1038/s41593-022-01186-3 PMC9630096 36303070

[45] Ji GJ Li J Liao W Wang Y Zhang L Bai T. Neuroplasticity-related genes and dopamine receptors associated with regional cortical thickness increase following electroconvulsive therapy for major depressive disorder. Mol Neurobiol (2023) 60:1465–75. doi: 10.1007/S12035-022-03132-7 36469225

[46] van Velzen LS Kelly S Isaev D Aleman A Aftanas LI Bauer J. White matter disturbances in major depressive disorder: a coordinated analysis across 20 international cohorts in the ENIGMA MDD working group. Mol Psychiatry (2019) 25:1511–25. doi: 10.1038/s41380-019-0477-2 PMC7055351 31471575

[47] Pang JC Aquino KM Oldehinkel M Robinson PA Fulcher BD Breakspear M. Geometric constraints on human brain function. Nature (2023) 2023:1–9. doi: 10.1038/s41586-023-06098-1 PMC10266981 37258669

[48] Lema YY Gamo NJ Yang K Ishizuka K. Trait and state biomarkers for psychiatric disorders: Importance of infrastructure to bridge the gap between basic and clinical research and industry. Psychiatry Clin Neurosci (2018) 72:482–9. doi: 10.1111/PCN.12669 29687938

[49] Zhang B Li S Zhuo C Li M Safron A Genz A. Altered task-specific deactivation in the default mode network depends on valence in patients with major depressive disorder. J Affect Disord (2017) 207:377–83. doi: 10.1016/J.JAD.2016.08.042 27750155

[50] Elkjær E Mikkelsen MB Michalak J Mennin DS O’Toole MS. Motor alterations in depression and anxiety disorders: A systematic review and meta-analysis. J Affect Disord (2022) 317:373–87. doi: 10.1016/J.JAD.2022.08.060 36037990

[51] Devaney KJ Levin EJ Tripathi V Higgins JP Lazar SW Somers DC. Attention and default mode network assessments of meditation experience during active cognition and rest. Brain Sci (2021) 11:566. doi: 10.3390/BRAINSCI11050566 33946661 PMC8144977

[52] Kong X Kong R Orban C Wang P Zhang S Anderson K. Sensory-motor cortices shape functional connectivity dynamics in the human brain. Nat Commun (2021) 12:1–15. doi: 10.1038/s41467-021-26704-y 34737302 PMC8568904

[53] Belkina AC Ciccolella CO Anno R Halpert R Spidlen J Snyder-Cappione JE. Automated optimized parameters for T-distributed stochastic neighbor embedding improve visualization and analysis of large datasets. Nat Commun (2019) 10:1–12. doi: 10.1038/s41467-019-13055-y 31780669 PMC6882880

[54] Tong Y Lindsey KP. Frederick BD. Partitioning of physiological noise signals in the brain with concurrent near-infrared spectroscopy and fMRI. J Cereb Blood Flow Metab (2011) 31:2352. doi: 10.1038/JCBFM.2011.100 21811288 PMC3253380

