# Supplementary material for: Manifold alteration between major depressive disorder and healthy control subjects using dynamic mode decomposition in resting-state fMRI data
Source: Front Psychiatry. 2024 Jan 30;15:1288808. doi: 10.3389/fpsyt.2024.1288808 (PMC10861746; doi:10.3389/fpsyt.2024.1288808)
Supplement: Supplementary file 1 [file DataSheet_1.docx]

# *Supplementary Material*

**Supplementary material regarding protocol and subject information**

**SI Table 1.** rsfMRI protocols in the SRPBS for training and another project dataset for testing COI: Center of Innovation at Hiroshima University; UTO: University of Tokyo; HKH: Hiroshima Kajikawa Hospital; HRC: Hiroshima Rehabilitation Center, Hiroshima University Hospital; UYA: Yamaguchi University.

**SI Table 2.** Subject statistics for each protocol. Numbers and male/female ratios are shown as counts. Age and BDI-Ⅱ are shown as mean and standard deviation, respectively. HCs, Healthy Controls, MDD: Major Depressive Disorder, COI: Center of Innovation in Hiroshima University, UTO: University of Tokyo, HKH: Hiroshima Kajikawa Hospital, HRC: Hiroshima Rehabilitation Center, Hiroshima University Hospital, UYA: Yamaguchi University

**Supplementary material regarding visualization method of manifold alteration between HCs and MDD**


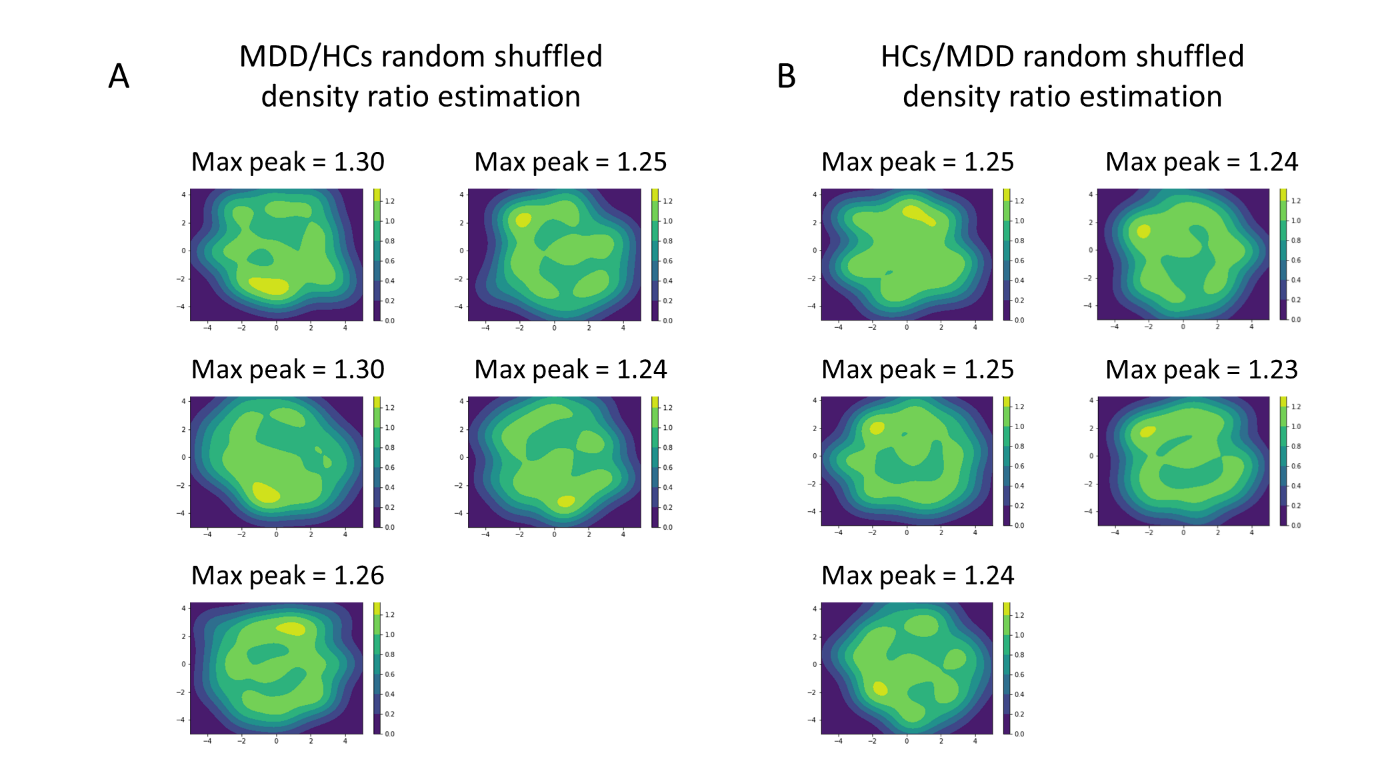


Figure S1. Density ratio distribution and the maximum peak values correspond to the 95th percentile for both MDD/HCs (A) and HCs/MDD (B) in the shuffled case. The following procedures were repeated 100 times to determine the threshold value for the 95% significance level. In the two-dimensional manifold using t-SNE, the class labels of all points representing HCs and MDD were randomly shuffled, and the maximum peak values were estimated using the relative unconstrained least-squares importance fitting (RuLSIF) method for both the MDD/HCs and HCs/MDD scenarios with fixed parameters $\left( \boldsymbol{\alpha, \sigma, \eta} \right)\boldsymbol{=(0, 1.0, 0.01)}$.


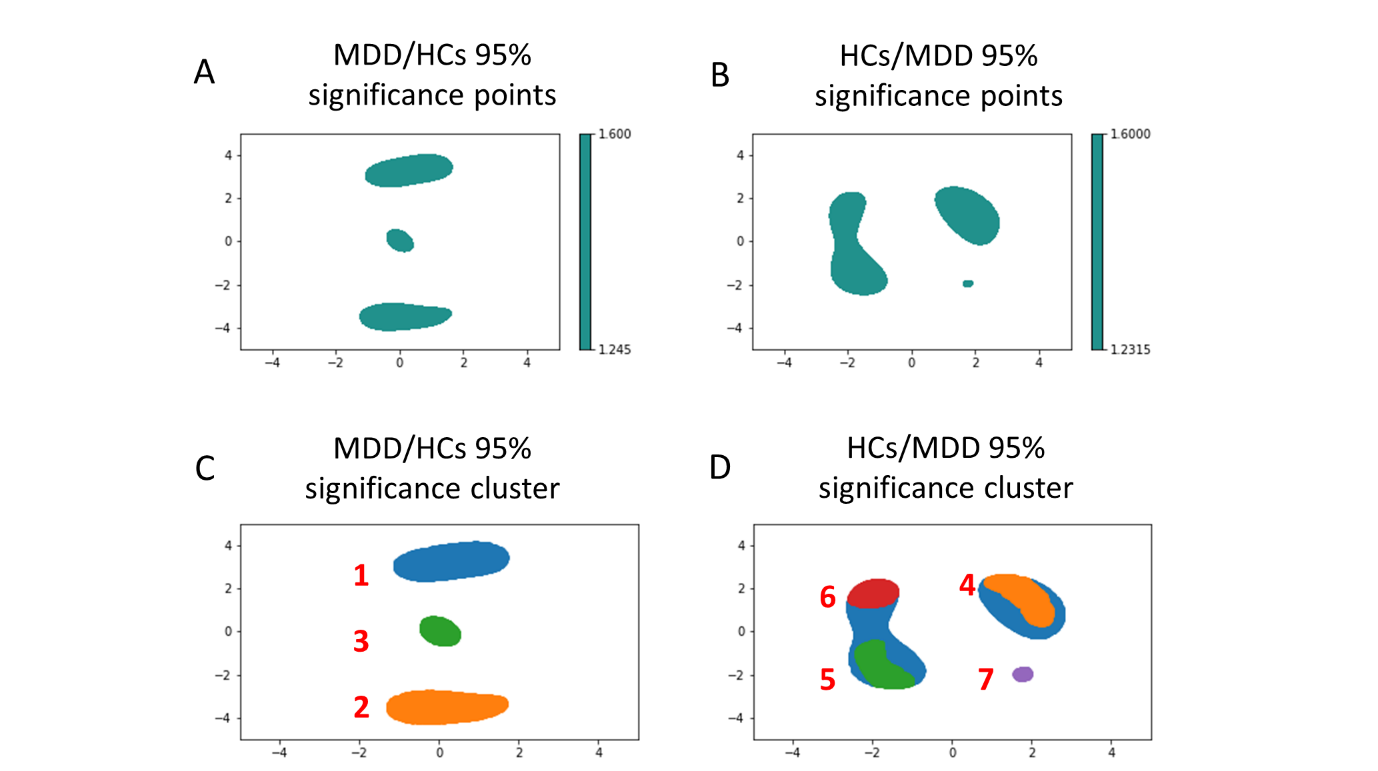


Figure S2. Areas exceeding the 95% significance level in the MDD/HCs (A) and HCs/MDD (B) and clusters in the MDD/HCs (C) and HCs/MDD (D) on the two-dimensional manifold. All data points that surpassed the 95% significance threshold on the two-dimensional manifold were selected. Subsequently, clustering was performed using density-based spatial clustering of applications with noise (DBSCAN) with parameter settings of $\left( \boldsymbol{eps, min samples} \right)\boldsymbol{=}\left( \boldsymbol{1, 100} \right)\mathbf{and}\boldsymbol{(0.15, 300)}$. Any data points that were not included within the identified clusters in D were excluded.


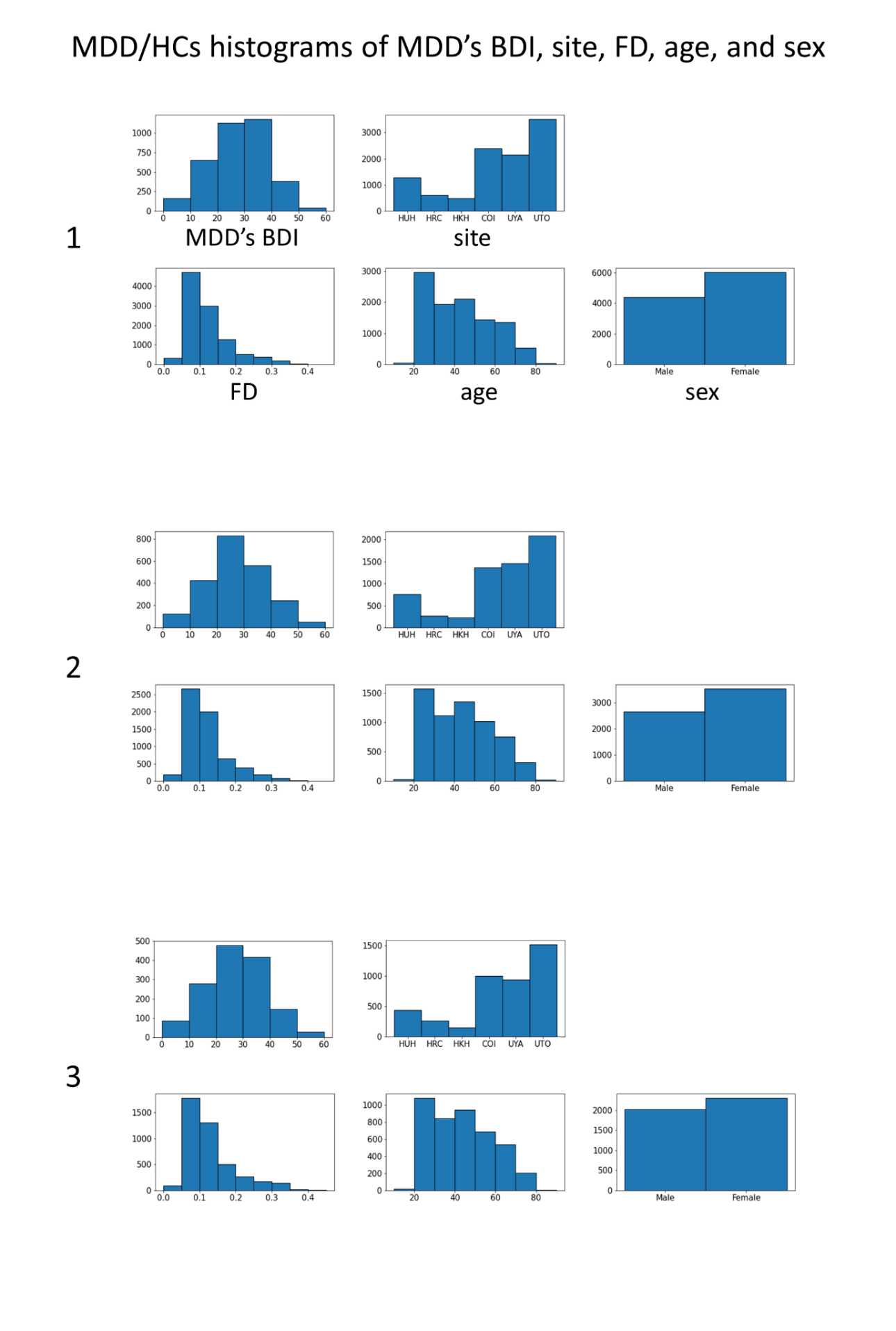


Figure S3. Histograms of Beck Depression Inventory-Ⅱ (BDI-Ⅱ) score, site, frame displacement (FD), age, and sex in each DM.


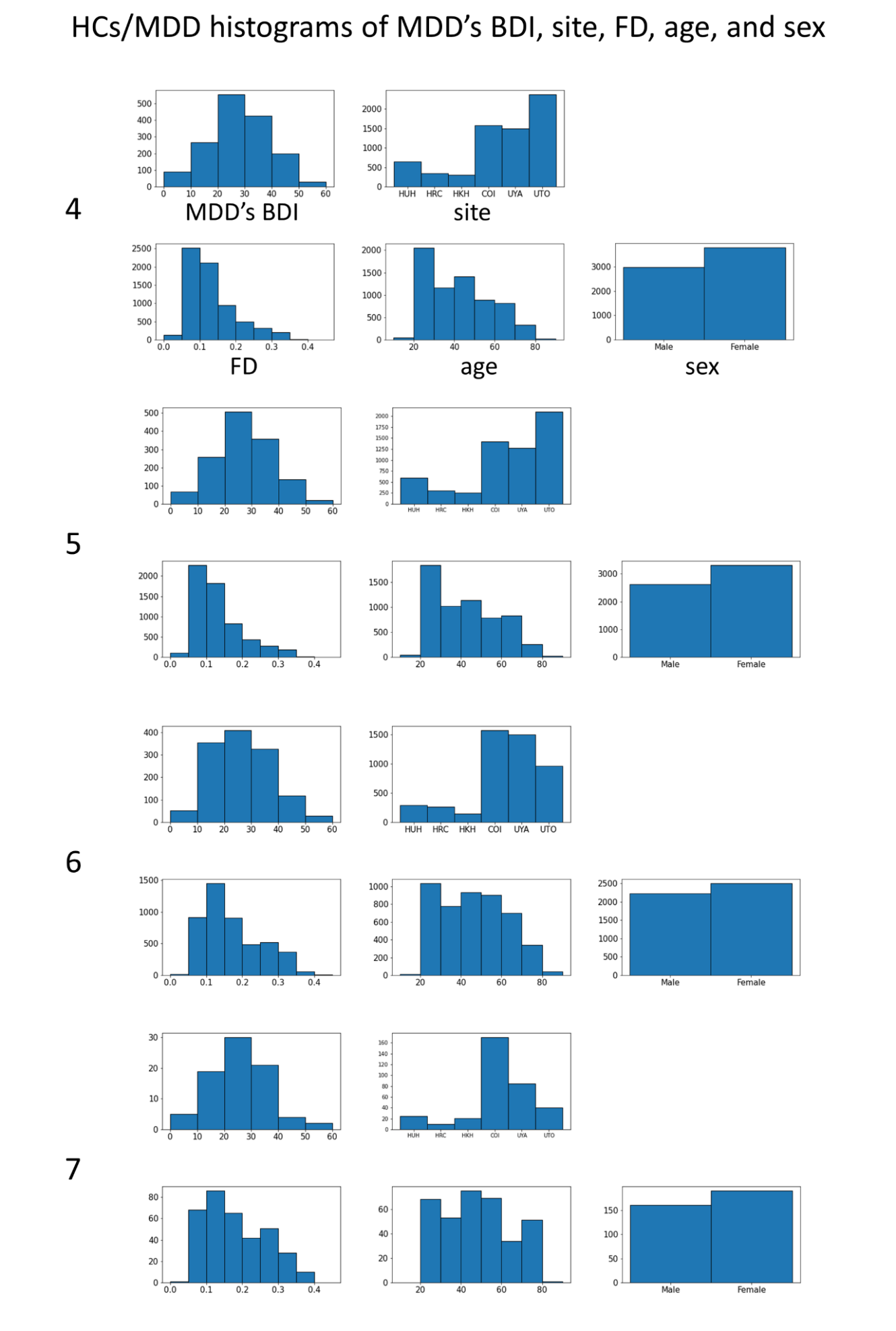


Figure S4. Histograms of BDI-Ⅱ score, site, FD, age, and sex in each DM.


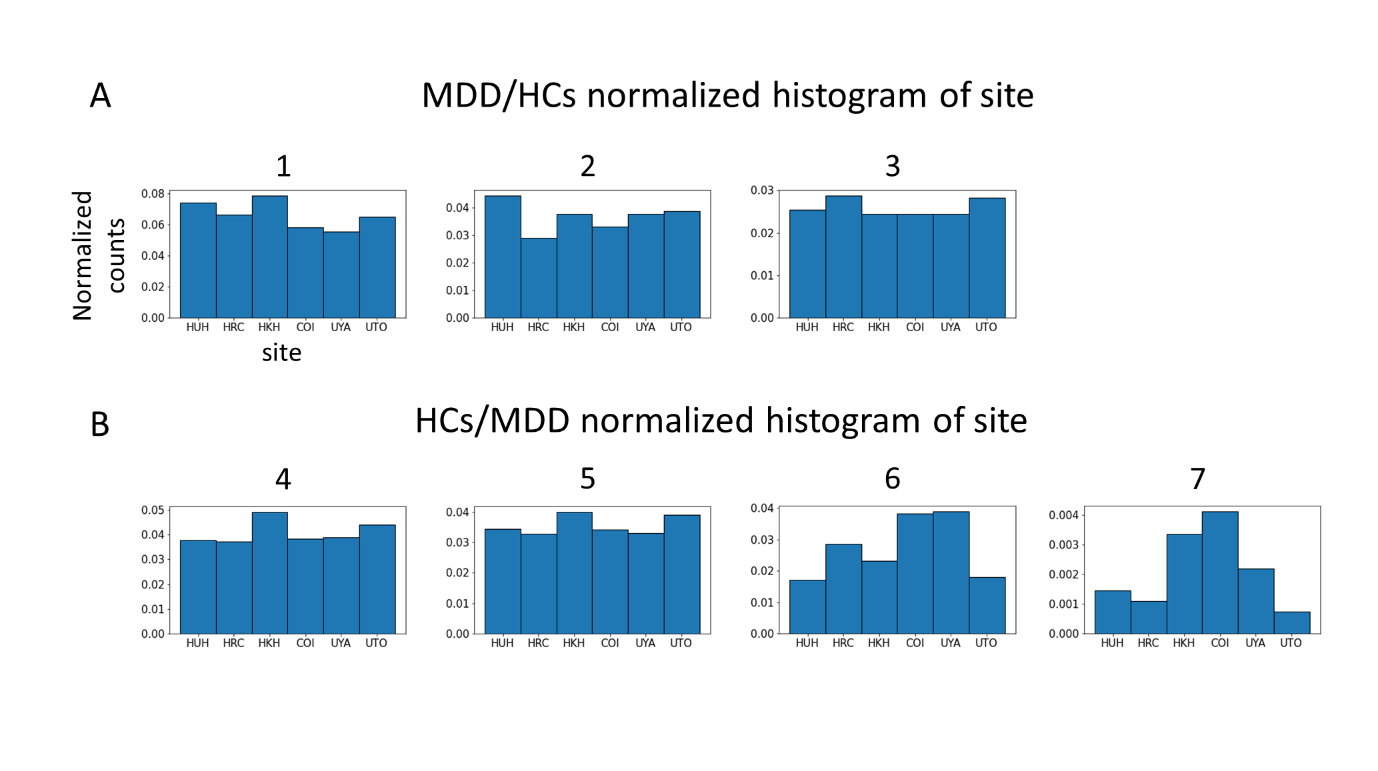


Figure S5. Normalized histogram of the site in the case of MDD/HCs (A) and HCs/MDD (B). The histogram values of the site were normalized by dividing the total number of DMs in the site because the total number of DMs in each site differed for protocol and the number of subjects in the site (SI Table 1, SI Table 2). In the case of DM1 to DM4, normalized counts between sites slightly differ. However, in the case of DM6, normalized counts are greatly different among sites, and UYAs are about 2.5 times higher than HUHs.

**Supplementary material regarding the prediction of HCs and MDD**


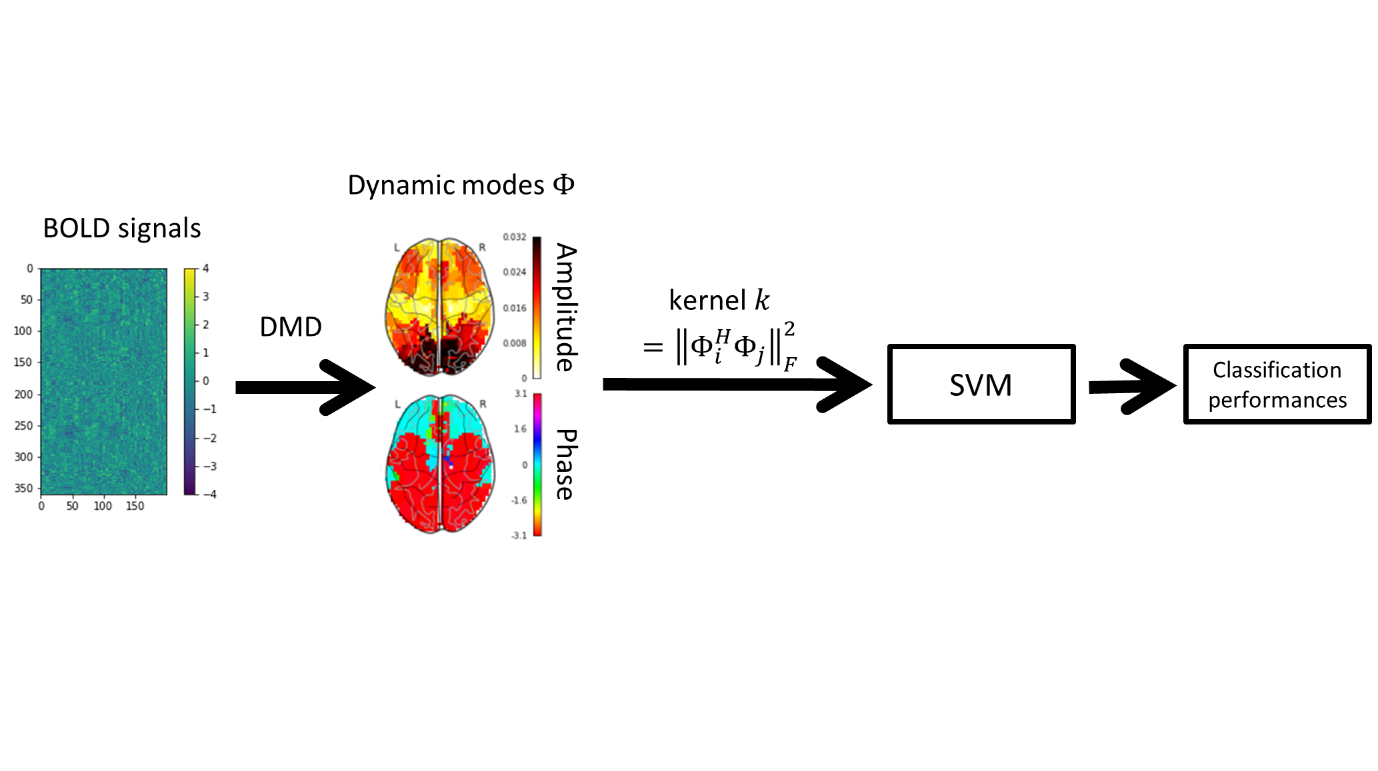


Figure S6. Overview of classification performance calculation. First, the blood oxygenation level-dependent (BOLD) signals from each subject were extracted using Glasser's 360 regions of interest (ROI). Second, the BOLD signals were decomposed into dynamic modes (DMs), and discrete-time eigenvalues using one-stacked time-delay coordinates dynamic mode decomposition (tdcDMD). Finally, metrics such as balanced accuracy (Bacc), Matthew's correlation coefficient (MCC), etc., were computed using a support vector machine (SVM) with Glassman kernels.

**Split training and test dataset**

The dataset shown in SI Table 2 was split for discriminant analysis (SI Table 3). COI and UTO with unified protocol were used for the training data set. HKH, HRC, HUH, and UYA with non-unified protocols were used for the test dataset.

In the training datasets, the total number of HCs and MDD was 280 and 121, respectively, with Beck Depression Inventory-Ⅱ (BDI-Ⅱ) scores of 8.0$\pm$6.3 and 23.8$\pm$10.3, respectively. In the test dataset, the total number of HCs and MDD was 263 and 181, respectively, with BDI-Ⅱ scores of 7.3$\pm$6.3 and 30.3$\pm$10.0, respectively.

SI Table 3. Subject statistics for each protocol. COI and UTO using unified protocol were used for training data. HKH, HRC, HUH, and UYA using non-unified protocols were used for testing data. Summary was calculated for each training and test dataset.

**Gram matrix composition**

Because of the absence of an inherent correspondence between subject-level DMs, a projection kernel was employed to classify HCs and MDD by considering the distance on the Grassmann manifold. Consistent with prior research (1), the projection kernel incorporated the amplitude of the DMs without orthonormalization. Building on previous studies (1–3), the formula for the projection kernel *k* is as follows:

$$k\left( \boldsymbol{\Phi}_{i}, \boldsymbol{\Phi}_{j} \right)=\left\| \boldsymbol{\Phi}_{i}^{*}\boldsymbol{\Phi}_{j} \right\|_{F}^{2},$$

where, $\boldsymbol{\Phi}_{i} \mathrm{and} \boldsymbol{\Phi}_{j}$ represent the DMs of subjects *i* and *j*, $\left\| \cdot\right\|_{F}$ represents Frobenius norm, and an asterisk represents Hermitian transpose.

Given that sFC was computed using BOLD signals within the frequency range of 0.01–0.08 Hz, the projection kernel was computed using DMs within the frequency range of 0.01–0.08 Hz, in addition to considering all frequencies to facilitate the comparison of prediction performance with sFC.

**Preprocess of ROI time series for sFC**

Physiological noise regressors were extracted using the CompCor software (4). To remove several sources of spurious variance, a linear regression with 12 regression parameters (six motion parameters, average signals over the entire brain, and five anatomical CompCor components) was applied. A temporal band-pass filter was applied to the time series using a second-order Butterworth filter with a pass band between 0.01 Hz and 0.08 Hz to restrict the analysis to low-frequency fluctuations, which are characteristic of rs-fMRI BOLD signals (5). This filtering process improved the classification performance compared to the use of all frequencies. The scrubbing process based on head motion was performed using frame displacement (FD) (6). FD was calculated using Nipype (<https://nipype.readthedocs.io/en/latest/>), and we removed volumes with FD > 0.5 mm, as proposed in a previous study (6). Using this threshold, 6.3% ± 13.5 volumes (mean ± SD) were removed per rsfMRI session in all datasets. Subjects whose ratio of excluded volumes by scrubbing exceeded the mean + 3 SD were excluded from the analysis. In summary, 401 participants (280 HCs and 121 patients with MDD) were included in the training dataset, and 444 participants (263 HCs and 181 patients with MDD) were included in the test dataset.

**sFC calculation**

After calculating the Pearson's correlation coefficient between BOLD signals using Glasser's 360 ROI, the sFC was calculated by applying Fisher's z-transformation. Following previous studies (7), a regression model utilizing a 1-of-K binary coding scheme was employed for harmonization purposes to mitigate the influence of different acquisition protocols on sFC.

For the training dataset, which included traveling subject data, traveling subject harmonization was applied to address the effect of the protocol on sFC. Conversely, for the test dataset, where traveling subject data were not available, ComBat harmonization (8) was applied to eliminate the effect of the protocol on sFC.

**Prediction with SVM both DMD and sFC**

A support vector machine (SVM) (9) was used to predict HCs and MDD in both DMD and sFC cases. Subsequently, six metrics were calculated: balanced accuracy (Bacc), sensitivity (Sen), specificity (Spec), positive predictive value (PPV), negative predictive value (NPV), and Matthews correlation coefficient (MCC).

In the case of DMD, 100 classifiers were initially generated by performing a 10-fold cross-validation (CV) repeated ten times on the training data. Then, by calculating the decision values of the remaining 1-fold using the 9-fold subsets within each CV iteration, the mean decision values across the CV folds were computed. The metrics were calculated by classifying HCs and MDDs based on the mean decision values. For the test data, HCs and MDD were initially predicted using the mean decision values calculated by the ten classifiers generated in each CV, and the mean and standard deviation of the metrics were calculated using the predictions from all CV iterations.

In the case of sFC, the same methodology as in a previous study (7) was employed. Initially, a 10-fold CV was performed on the training data. For a specific 9-fold combination, MDD data were randomly subsampled, and an equal number of HCs were subsampled to minimize age differences as much as possible. This process was repeated ten times, resulting in the generation of ten classifiers. The mean decision values of the remaining 1-fold were then calculated using the SVM with these ten classifiers. Finally, this procedure was repeated ten times for all 9-fold combinations, and the metrics were computed accordingly. For the test data, HCs and MDD were predicted based on the mean decision values obtained from all the classifiers, and the metrics were calculated accordingly.


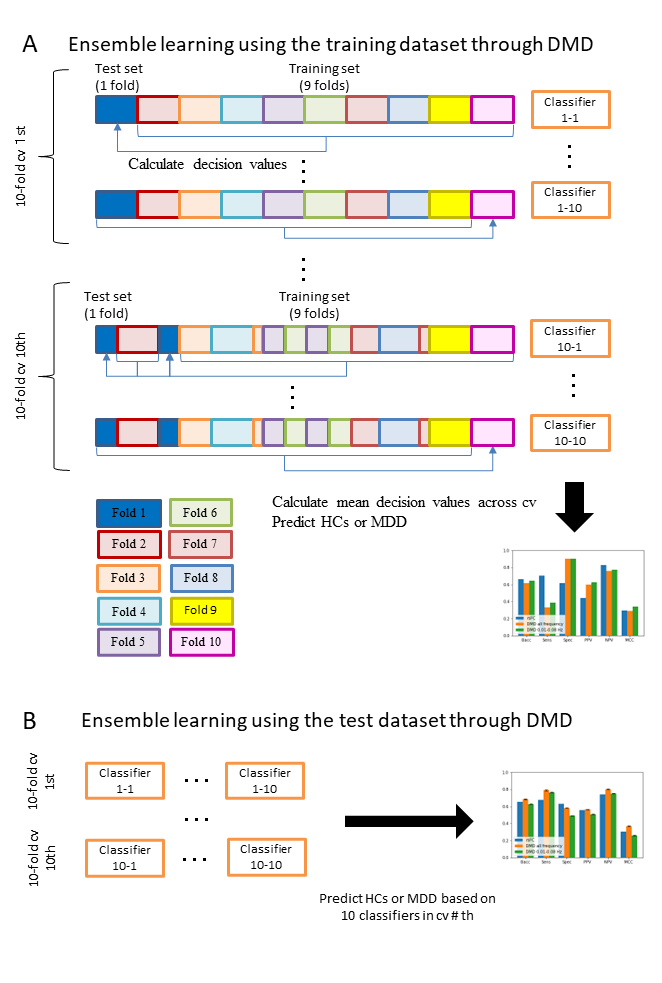


Figure S7. Overview of the ensemble learning procedure in the training dataset (A) and test dataset (B) using DMD. During the training phase, 10-fold cross-validation was repeated ten times, resulting in the generation of 100 classifiers. Predictions for HCs and MDD were made based on the mean decision values. In the test phase, predictions for HCs or MDD were made using the mean decision values obtained from each 10-fold cross-validation.

**MDD classifier performances using DMD and sFC**

The metrics derived from sFC and DMD are shown in Figure S8. Bacc, Sen, Spec, PPV, NPV, and MCC represent the balanced accuracy, sensitivity, specificity, positive predictive value, negative predictive value, and Matthew’s correlation coefficient, respectively.

In the order of DMD (all frequency), DMD (0.01–0.08 Hz), and sFC (0.01–0.08 Hz), the training dataset yielded Bacc values of approximately 0.617, 0.644, and 0.660, while the corresponding Bacc values in the test dataset were approximately 0.685, 0.627, and 0.654. The Bacc values obtained from sFC were consistent with the findings of a previous study (10). In both cases of DMD (0.01–0.08 Hz) and sFC (0.01–0.08 Hz), Bacc in the training dataset exceeded that of DMD (all frequencies). However, in the test dataset, the highest Bacc was observed for DMD (all frequencies), while Bacc was lower for DMD (0.01–0.08 Hz) and sFC (0.01–0.08 Hz) compared to the training dataset. The BDI-Ⅱ scores for HCs and MDD in the training dataset were 8.0 ± 6.3 and 23.8 ± 10.3, respectively, while in the test dataset, the scores were 7.3 ± 6.3 and 30.3 ± 10.0. Consequently, despite the expectation that Bacc would be easier to classify in the test dataset than in the training dataset, it slightly decreased. In other words, overlearning occurred in both cases of DMD (0.01–0.08 Hz) and sFC (0.01–0.08 Hz). However, Bacc increased in the case of DMD (all frequencies).


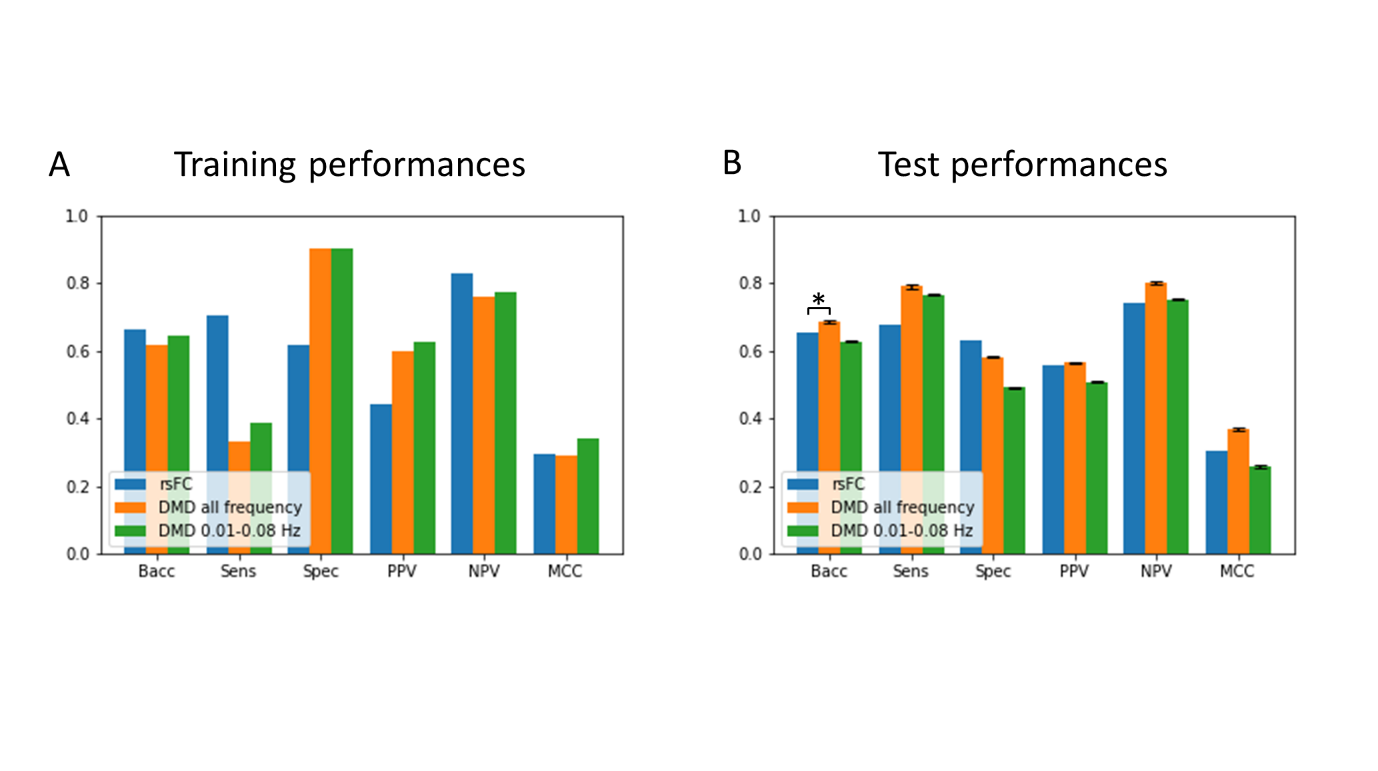


Figure S8. Metrics of training (left) and test (right) dataset using sFC (0.01–0.08 Hz), DMD (all frequencies), and DMD (0.01–0.08 Hz). Color bars in the graph correspond to the lower left color bars and their corresponding names. An asterisk indicates significance. Bacc, balanced accuracy; Sen, sensitivity; Spec, specificity; PPV, positive predictive value; NPV, negative predictive value; MCC, Matthew’s correlation coefficient.

**References**

1. Ikeda S, Kawano K, Watanabe S, Yamashita O, Kawahara Y. Predicting behavior through dynamic modes in resting-state fMRI data. *Neuroimage* (2022) 247:118801. doi: 10.1016/J.NEUROIMAGE.2021.118801

2. Hamm J, Lee DD. Grassmann discriminant analysis: A unifying view on subspace-based learning. *Proceedings of the 25th International Conference on Machine Learning*. (2008)

3. Shiraishi Y, Kawahara Y, Kawahara Y, Yamashita O, Yamashita O, Fukuma R, Fukuma R, Yamamoto S, Saitoh Y, Saitoh Y, et al. Neural decoding of electrocorticographic signals using dynamic mode decomposition. *J Neural Eng* (2020) 17: doi: 10.1088/1741-2552/ab8910

4. Behzadi Y, Restom K, Liau J, Liu TT. A component based noise correction method (CompCor) for BOLD and perfusion based fMRI. *Neuroimage* (2007) 37:90–101. doi: 10.1016/J.NEUROIMAGE.2007.04.042

5. Ciric R, Wolf DH, Power JD, Roalf DR, Baum GL, Ruparel K, Shinohara RT, Elliott MA, Eickhoff SB, Davatzikos C, et al. Benchmarking of participant-level confound regression strategies for the control of motion artifact in studies of functional connectivity. *Neuroimage* (2017) 154:174–187. doi: 10.1016/J.NEUROIMAGE.2017.03.020

6. Power JD, Mitra A, Laumann TO, Snyder AZ, Schlaggar BL, Petersen SE. Methods to detect, characterize, and remove motion artifact in resting state fMRI. *Neuroimage* (2014) 84:320–341. doi: 10.1016/J.NEUROIMAGE.2013.08.048

7. Yamashita A, Sakai Y, Yamada T, Yahata N, Kunimatsu A, Okada N, Itahashi T, Hashimoto R, Mizuta H, Ichikawa N, et al. Generalizable brain network markers of major depressive disorder across multiple imaging sites. *PLoS Biol* (2020) 18:e3000966. doi: 10.1371/JOURNAL.PBIO.3000966

8. Yu M, Linn KA, Cook PA, Phillips ML, McInnis M, Fava M, Trivedi MH, Weissman MM, Shinohara RT, Sheline YI. Statistical harmonization corrects site effects in functional connectivity measurements from multi-site fMRI data. *Hum Brain Mapp* (2018) 39:4213–4227. doi: 10.1002/HBM.24241

9. Chang CC, Lin CJ. LIBSVM. *ACM Transactions on Intelligent Systems and Technology (TIST)* (2011) 2: doi: 10.1145/1961189.1961199
